# The Wandering Gallbladder: A Rare Case of Acute Cholecystitis in the Falciform Ligament

**DOI:** 10.7759/cureus.93255

**Published:** 2025-09-26

**Authors:** Jacob Clark, Armein Rahimpour, Karim Abdelgaber, Jentre H Hyde, Aishwarya S Vijay, Paul Bown

**Affiliations:** 1 General Surgery, Marshall University Joan C. Edwards School of Medicine, Huntington, USA; 2 Surgery, Marshall University Joan C. Edwards School of Medicine, Huntington, USA

**Keywords:** acute cholecystitis, anatomical variation, ectopic gallbladder, falciform ligament, gallbladder anomaly

## Abstract

Ectopic gallbladders are uncommon congenital variants that can complicate management of acute cholecystitis. They are rare, and their unusual positioning can pose diagnostic and intraoperative challenges. Because of their rarity, many ectopic gallbladders are not suspected until the time of surgery, increasing the potential risk of bile duct injury and prolonging operative time. Here, we present the case of a 41-year-old woman presenting with severe overnight epigastric pain following six months of intermittent pain. Examination revealed right upper quadrant and epigastric tenderness with a positive Murphy’s sign. Liver function tests were within normal limits, and imaging demonstrated cholelithiasis with mural edema and a positive sonographic Murphy’s sign. The patient underwent a laparoscopic cholecystectomy, where the patient’s gallbladder was found to arise from the falciform ligament, with further exploration showing no malrotation. The critical view of safety was achieved, the cystic artery and duct were clipped and divided, and the procedure was completed without hemorrhage or bile leak. The patient was discharged on postoperative day one. This case highlights the importance of maintaining awareness of rare anatomic variants to avoid bile duct injury and demonstrates that laparoscopic cholecystectomy remains a safe and effective approach in the presence of such anomalies.

## Introduction

The gallbladder is most commonly located in the gallbladder fossa on the visceral surface of the right hepatic lobe. There can be various rare congenital anomalies that result in the gallbladder being located in sites including intrahepatic, retrohepatic, transverse, or left-sided [1]. These variations arise from aberrant migration of the hepatic diverticulum during embryogenesis, leading to displacement of the gallbladder from its usual position [2]. During the fourth week of development, the hepatic diverticulum divides into the pars hepatica and pars cystica; abnormal budding, migration, or fusion of the pars cystica can explain these rare ectopic positions [2]. While there are growing reports of left-sided and other anomalously located gallbladders, there are very few reports of gallbladders within or originating from the falciform ligament [3,4]. Such anomalies present unique challenges intraoperatively if unanticipated. Ectopic gallbladders have also been associated with complications such as torsion, delayed diagnosis, and difficulty in radiologic identification, further emphasizing their clinical relevance. Here, we present a case of an acute gallbladder arising from the falciform ligament, managed successfully with laparoscopic cholecystectomy.

## Case presentation

The patient was a 41-year-old female who presented to the emergency department with severe epigastric pain beginning overnight; it was dull, radiating to her back, and 9/10 in intensity. The patient had been experiencing similar pain for six months intermittently; however, overnight, the pain became more intense than the patient had experienced previously. On physical exam, the patient was vitally stable and afebrile. The abdomen was found to be tender to palpation in the right upper quadrant and epigastric region without rebound and with a positive Murphy’s sign. No masses or rigidity were noted. The patient had a history of hysterectomy with unilateral oophorectomy three years prior. Past medical history was significant for gastroesophageal reflux disease (GERD) and morbid obesity (BMI 34). She denied alcohol use, cigarette smoking, or illicit drugs. Denies any family history of hepatobiliary pathology. The patient is not on estrogen therapy or weight loss medication.

**Figure 1:**
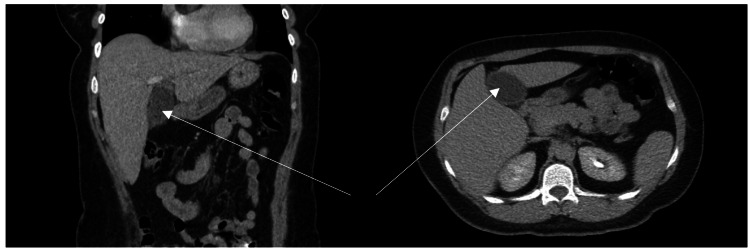
CT scan of the abdomen and pelvis Cholelithiasis with a mildly prominent gallbladder wall is observed. The arrows point to the gallbladder.

**Figure 2:**
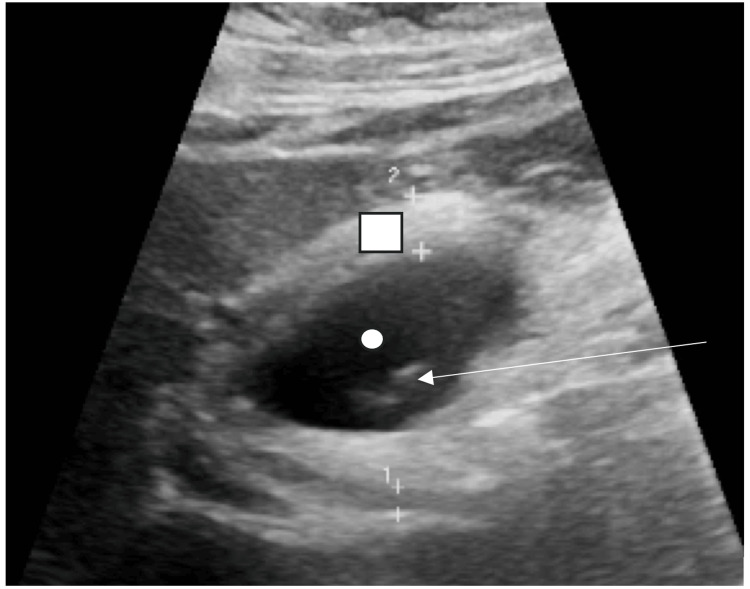
UItrasound of the right upper quadrant White circle: Gallbladder, Arrow: Cholelithiasis, Square: Gallbladder wall thickening

A CT of the abdomen and pelvis on admission showed cholelithiasis with a mildly prominent gallbladder wall (Figure 1). Ultrasonography of the right upper quadrant showed cholelithiasis with gallbladder mural edema and a positive sonographic Murphy’s sign, consistent with acute cholelithiasis (Figure 2). Her laboratory work-up demonstrated normal bilirubin, transaminases, alkaline phosphatase, and white blood cell count, suggesting no concurrent choledocholithiasis or cholangitis. Based on these findings, the patient was scheduled for a laparoscopic cholecystectomy the following day.

**Figure 3:**
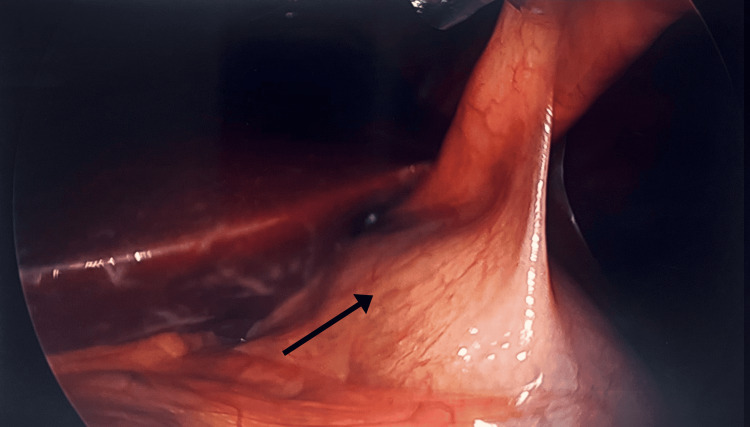
Intraoperative laparoscopic view showing an ectopic gallbladder arising from the falciform ligament The gallbladder is seen abnormally positioned, suspended within the falciform ligament (black arrow), with associated inflammatory changes. This rare anatomical variant poses significant implications for surgical identification and dissection during cholecystectomy.

During the procedure, pneumoperitoneum was established with a Veress needle in the upper left quadrant, and a 12 mm trocar was placed at the umbilicus using the Hasson technique. Inspection revealed the gallbladder arising from and partially embedded within the falciform ligament (Figure 3). This unusual positioning initially obscured Calot’s triangle until retraction was carefully optimized. Three additional 5 mm trocars were also placed in the right upper quadrant in the usual standard fashion. The rest of the abdomen was then explored, and no evidence of malrotation or any other anatomical anomaly was found. After retracting the gallbladder fundus cephalad, Calot’s triangle was dissected, and the critical view of safety was achieved. The cystic artery was identified and clipped proximally twice and distally once, and divided. Likewise, the cystic duct was identified and clipped proximally twice and distally once, and divided. The gallbladder was removed from the liver bed using Bovie cautery before being placed in an endocatch bag and extracted through the umbilical port. Hemostasis was confirmed, and there was no bile leakage. All instruments and ports were recovered from the abdomen. 

The total operative time was 37 minutes with an estimated blood loss of <10 mL. The patient’s postoperative course was uncomplicated, with early ambulation, toleration of oral intake, and pain adequately controlled with acetaminophen and non-opioid analgesics. The patient was discharged on postoperative day one and reported complete resolution of symptoms at her one-week follow-up visit. Pathology demonstrated acute and chronic cholecystitis with cholesterolosis and cholelithiasis.

## Discussion

Ectopic gallbladders, which can be found in various locations, are exceedingly rare, with incidences reported to be below 0.7% [5,6]. The most common ectopic gallbladder location is beneath the left lobe of the liver, called a left-sided gallbladder [7]. Other reported sites include intrahepatic, retroperitoneal, transverse mesocolon, and within the abdominal wall. Normally, the gallbladder develops from the pars cystica of the hepatic diverticulum. Its usual location is in the right upper quadrant, lying in the gallbladder fossa on the visceral (inferior) surface of the right hepatic lobe, between hepatic segments IVB and V. Abnormal migration or failure of rotation can explain ectopic positioning, including attachment to the falciform ligament. Ectopic gallbladders may lead to diagnostic uncertainty and misinterpretation of imaging studies. Furthermore, this could cause difficulties with a typical cholecystectomy.

There are very few reports of gallbladders arising from the falciform ligament as presented in this report. In fact, fewer than a dozen cases have been described in the English language literature, underscoring the rarity of this anomaly. Though rare, it is important to be aware of the possibility of an ectopic gallbladder when preparing for surgical intervention or interpreting right upper quadrant imaging. Ultrasound, while widely used, may fail to identify ectopic positioning due to limitations in acoustic windows, and CT can similarly be misleading if the gallbladder is not in its expected fossa. Magnetic resonance cholangiopancreatography (MRCP) is the most reliable noninvasive modality [8] for delineating biliary anatomy and should be considered when anomalous anatomy is suspected.

Management remains laparoscopic cholecystectomy even for anomalously positioned gallbladders [9], though intraoperative strategies may require modification for the discovered anatomy. Some reports describe altered port placement and division of the falciform depending on the altered anatomy [10], with heavy reliance on both preoperative and intraoperative imaging. In our case, conventional port placement sufficed, but had the gallbladder been more deeply embedded in the falciform ligament, additional port placement or an open procedure might have been necessary for exposure and removal.

In our case, the anomalous position of the gallbladder was only recognized intraoperatively, but the major anatomical landmarks were discernible without much difficulty. In many cases of ectopic gallbladders, the anatomy can be distorted, making careful dissection difficult. Difficulty visualizing or accessing the anatomy can necessitate conversion to an open approach or intraoperative cholangiography to better identify the biliary tree and associated structures [9]. Obtaining the critical view of safety is essential to minimizing the risk of bile duct injury. Other authors [11] have emphasized the value of a low threshold for cholangiography in ectopic cases, as bile duct anomalies are frequently associated

This case adds to the limited body of evidence supporting laparoscopic management of gallbladders arising from the falciform ligament. It also highlights the need for collaboration between radiologists and surgeons in recognizing unusual gallbladder positions, as preoperative recognition can prevent operative confusion. Furthermore, accumulating and publishing such cases will eventually allow for clearer surgical guidelines in dealing with these anomalies.

## Conclusions

A gallbladder arising from the falciform ligament is an exceptionally rare ectopic gallbladder variant. Surgeons should be aware of such anomalies and be prepared to adapt management strategies when surgical management is required. Careful dissection and obtaining the critical view of safety allow for successful laparoscopic management without complications. This report emphasizes that while standard surgical principles apply, heightened vigilance and flexibility in operative strategy are necessary when encountering ectopic biliary anatomy. Further reporting of similar cases will enhance collective understanding and improve outcomes for this rare entity.
